# A distinct ripple-formation regime on Mars revealed by the morphometrics of barchan dunes

**DOI:** 10.1038/s41467-022-34974-3

**Published:** 2022-11-22

**Authors:** Lior Rubanenko, Mathieu G. A. Lapôtre, Ryan C. Ewing, Lori K. Fenton, Andrew Gunn

**Affiliations:** 1 Department of Geological Sciences, Stanford University, Stanford, CA 94305 USA; 2 Department of Geology & Geophysics, Texas A&M, College Station, TX 77843 USA; 3 Carl Sagan Center, SETI Institute, Mountain View, CA 94043 USA; 4 School of Earth, Atmosphere & Environment, Monash University, Clayton, VIC 3800 Australia

**Keywords:** Geomorphology, Geomorphology

## Abstract

Sand mobilized by wind forms decimeter-scale impact ripples and decameter-scale or larger dunes on Earth and Mars. In addition to those two bedform scales, orbital and in situ images revealed a third distinct class of larger meter-scale ripples on Mars. Since their discovery, two main hypotheses have been proposed to explain the formation of large martian ripples—that they originate from the growth in wavelength and height of decimeter-scale ripples or that they arise from the same hydrodynamic instability as windblown dunes or subaqueous bedforms instead. Here we provide evidence that large martian ripples form from the same hydrodynamic instability as windblown dunes and subaqueous ripples. Using an artificial neural network, we characterize the morphometrics of over a million isolated barchan dunes on Mars and analyze how their size and shape vary across Mars’ surface. We find that the size of Mars’ smallest dunes decreases with increasing atmospheric density with a power-law exponent predicted by hydrodynamic theory, similarly to meter-size ripples, tightly bounding a forbidden range in bedform sizes. Our results provide key evidence for a unifying model for the formation of subaqueous and windblown bedforms on planetary surfaces, offering a new quantitative tool to decipher Mars’ atmospheric evolution.

## Abstract (web summary)

Dust storms on Mars drive water escape to space. Here, the authors show the impact Martian dust storms have on the abundance of atmospheric hydrogen and oxygen, and how this helps to overall oxidize the Martian atmosphere.

## Introduction

**Fig. 1:**
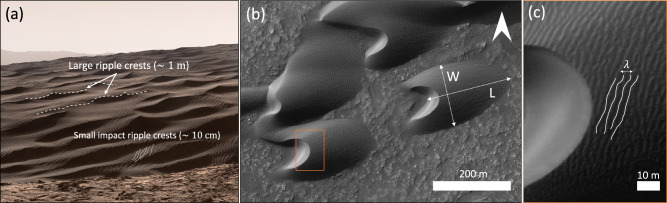
Dunes and ripples on Mars. **a** Impact ripples superimposed on large wind ripples atop Namib dune, Gale Crater, Mars. (Curiosity rover Mastcam mosaic; credit: NASA/JPL-Caltech/MSSS/Thomas Appéré). **b** Large ripples superimpose barchan dunes, Hellespontus Montes, Mars (−41.4°N, 44.6°E; High Resolution Imaging Science Experiment (HiRISE) image PSP_007676_1385). **c** Detailed view of large ripples with wavelength ~2 m (orange rectangle in panel (**b**)).

From decimeter-scale ripples to decameter-scale or larger dunes, aeolian (windblown) bedforms are ubiquitous across Mars’ desert landscapes. In addition to small-scale ripples, a distinctly larger class of meter-scale ripples was discovered on Mars from observations made by NASA’s Curiosity rover^1,2^ (Fig. 1). On Earth, meter-scale ripples only form in the presence of wide or bimodal grain-size distributions, with two grain-size populations moving through two distinct transport modes—saltation and creep^3^. However, many large ripples on Mars lack a concentration of coarser grains that would be transported in creep near their crests^4–8^ and thus cannot be explained by bimodal transport^3^.

Two models have been proposed to date to explain the co-existence of the two distinct ripple scales on Mars. Under the first model, large ripples would result from the continuous growth of small ripples from saltation-driven impact splash, enabled by low dynamic wind pressures on Mars (i.e., they would be impact ripples^9^). However, ripples with intermediate sizes (~20–80 cm) were not observed to be active in relatively well sorted sand^1,2,5,6,10^, contradicting the model’s prediction that a continuum of ripple sizes should exist between those of small and large ripples. Furthermore, the crest-to-crest spacing (or wavelength, *λ*, Fig. 1) of large martian ripples was shown to decrease with increasing atmospheric density (*ρ*_*f*_), following a trend that is quantitatively consistent with predictions from a scaling relationship developed for subaqueous drag ripples^1,5,11^. Consistent with those observations, another model^12^ suggests that large wind ripples form from the same hydrodynamic instability^13^ as windblown dunes and subaqueous bedforms. Specifically, the scale separation between Mars’ windblown large ripples and dunes (and between subaqueous ripples and dunes) would arise from a hydrodynamic anomaly^14,15^ that prevents the growth of bedforms with intermediate wavelengths^12^.

Under hydrodynamically smooth flow conditions, a hydrodynamic anomaly (hereafter referred to as the Hanratty anomaly) was shown to arise as bedform size increases^14,15^. This anomaly is linked to the way pressure gradients are balanced within the inner boundary layer in response to bedform topography; it arises when the flow within the inner boundary layer (disturbed by bedform topography) remains laminar upstream of the bedform crest but becomes turbulent downstream as turbulent fluctuations amplify^12^. The anomaly thus depends on the turbulent response imparted by the bedform and takes place as the disturbed flow in the inner boundary layer transitions from fully laminar to fully turbulent across the bedform length, as has also been proposed for drag bedforms^11^. This transitional regime is defined by a range in bedform-scale Reynolds number, ${{{{{{\rm{Re}}}}}}}_{ \lambda }=\frac{ \lambda {u}_{*}}{\nu }$ (with $\lambda$ the bedform wavelength, ${u}_{*}$ the wind shear velocity, and $\nu$ the flow’s kinematic viscosity), which may vary with sediment transport conditions^11,12^. This hydrodynamic anomaly was reported from a single set of well controlled experiments where the bed, unlike natural dunes, was non-erodible^14,15^; however, its existence has not yet been demonstrated in nature where bed topography arises from flow-driven sediment transport and deposition.

Existing theory for the hydrodynamic instability that generates dunes is formulated for ideal transverse (i.e., migrating roughly perpendicularly to average crestline orientation) periodic dunes with unlimited sediment supply. Barchans dunes are transverse bedforms that form under relatively unimodal winds, but they occur under limited sediment supply^16^. However, the size of nascent barchan dunes, approximated here as the smallest dunes in their immediate environment (2nd percentile in bins of size 60 km^2^), is expected to be similar to the wavelength of incipient periodic transverse dunes that would arise under the same flow conditions but greater sediment supply^17^. Thus, because they are prevalent across Mars^18–22^ and are ideal objects for machine-learning assisted detection due to their unique isolated crescentic shape (Methods), they offer a unique opportunity to investigate the environmental controls on dune sizes and shapes (Fig. 1b) across Mars’ surface as a test to bedform-formation theory^12^. Specifically, if Mars’ large ripples and dunes form from the same hydrodynamic instability, both the wavelength of large ripples (as was evidenced from orbital imagery^1^) and the size of the smallest barchan dunes should decrease with increasing atmospheric density following a similar trend. In turn, the observed decrease in bedform wavelength should be characterized by a power-law exponent strictly greater than −1 ($\lambda \propto {\rho }_{f}^{-1}$ for barchans whose length, like on Earth, scales with transport saturation length ^23–25^, the spatial scale over which sand flux equilibrates to its saturated value) and smaller or equal to −1/2 (i.e., bedform size is influenced by the viscous length scale, $\frac{\nu }{{u}_{*}}$; $\lambda \propto {\rho }_{f}^{-1/2}$ for sand transport at threshold conditions; Methods)^11^.

**Fig. 2:**
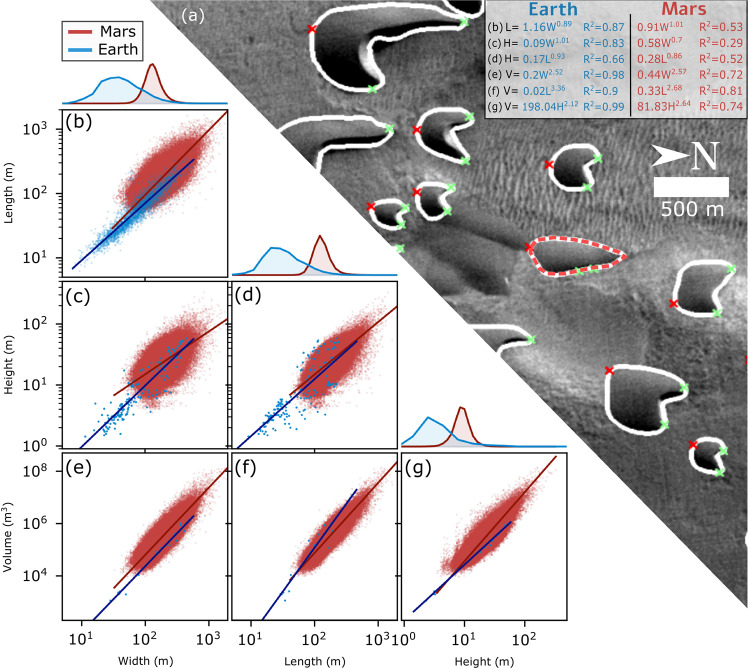
Morphometrics of barchan dunes on Mars. **a** Example of automatically detected barchans on Mars (−42.20°N, −31.89°E). Outlines (white) were identified by the neural network in Mars Reconnaissance Orbiter (MRO) Context Camera (CTX)^42^ images. The red dashed outline shows a detection that was discarded by our algorithm on the basis of the absence of a convexity deffect. Red and green crosses indicate the automatically detected tails and horns, respectively. **b**–**g** Correlations between morphometric parameters on Mars (red) and Earth (blue). Line histograms indicate the marginal distribution of each parameter. Regression lines are shown as red (Mars) and blue (Earth) lines; *R*^2^ values were computed as the square of Pearson’s correlation coefficient.

In this work, we test this hypothesis by generating a global dataset of barchan morphometrics (Figs. S1–S2) on Mars to reveal geospatial trends as they relate to the global near-surface atmospheric circulation and local environmental conditions. Our extensive dataset, which includes over a million barchan dunes previously outlined by an instance segmentation neural network and validated using a comprehensive manually processed dataset^26^ (Fig. 2a and Figs. S3–S5), reveals the size of barchan dunes on Mars decreases with increasing atmospheric density, similar to the wavelength of large ripples. Our results support the hypothesis^1,12^ that large ripples on Mars form from the same hydrodynamic instability as dunes, indicating that ancient aeolian bedforms can be used to reconstruct the density of Mars’ atmosphere at the time of sediment deposition.

## Results

### Global morphometrics of barchan dunes on Mars

We find that the relationships between dune width (W), length (L), height (H) and volume (V; Fig. 2b–g) on Mars closely follow terrestrial scaling laws. Dune length and width display a near-linear relationship ($L\propto {W}^{1.011\pm 0.001}$, ${R}^{2}=0.52$, $p \, < \, 0.01$). Our estimates of dune height, which are derived from imagery and are sensitive to illumination, show greater scatter but remain close to linear functions of planform dune dimensions. A stronger correlation between dune length and height hints that dune length is a more robust predictor of height than width, which shows greater scatter ($H\propto {L}^{0.858\pm0.001},\,{R}^{2}=0.52,\,{p} \, < \, 0.01{;\; H}\propto {W}^{0.702\pm0.004},\,{R}^{2}=0.29,\,{p} \, < \, 0.01$). Correlations between the planform dimensions of barchans and their estimated volumes show relatively low scatter. When fitted with a power law, dune volume follows a near-cubic relationship with both width and length ($V\propto {W}^{2.577\pm 0.001},{R}^{2}=0.72,\,{p} \, < \, 0.01$ and $V\propto {L}^{2.669\pm 0.002},\,{R}^{2}=0.81,\,{p} \, < \, 0.01$). We find that barchan dunes on Mars and Earth follow similar linear relationships between length and width ($L\propto 0.9W$), height and length $(H\propto 0.11L)$, and height and width ($H\propto 0.08W$)^27,28^, and follow cubic scaling laws of $V\sim \frac{1}{37}{L}^{3}$ and $V\sim \frac{1}{42}{W}^{3}$, which are similar to barchan dunes on Earth^28^. These similarities between barchan scaling laws previously derived on Earth to those we derived using our comprehensive dataset on Mars demonstrates that dunes on Mars and Earth share important morphodynamics^29^ and could be used to constrain general physical laws of aeolian transport on both planets.

**Fig. 3:**
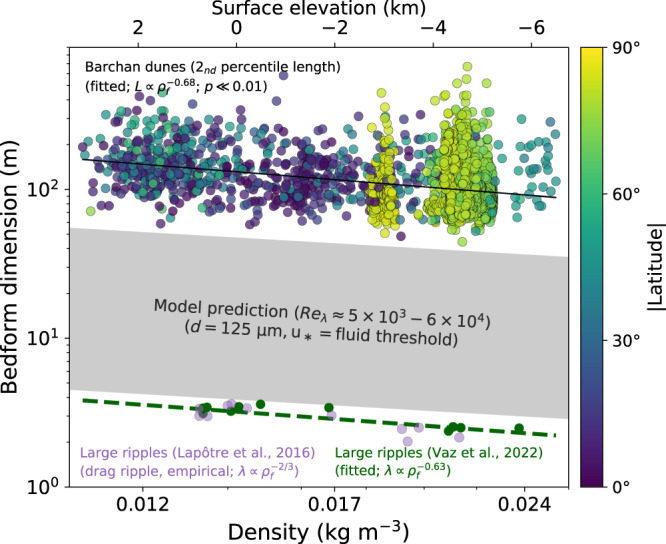
Dune length (blue-to-yellow circles) and large-ripple wavelength (light purple^1^ and dark green circles^36^) decreases with increasing atmospheric density. Ripple data follows a previously derived scaling relationships for drag ripples^5^; best-fit power law to an updated global compilation of dark-toned large-ripple wavelength has an exponent of −0.63 (green dashed line)^35^. The width of the hydrodynamic anomaly (gray shade) was calculated for a 125 μm grain size^7^ and assuming that wind shear velocity is at the transport threshold^49^ (Methods), using the model of ref. 12. The statistical significance of the regression slope was computed using a Wald Test, and errorbars (narrower than line width) indicate a 95% confidence interval. Each dot in the figure represents the 2nd percentile dune length in equal-area bins (60 km^2^).

Furthermore, we find that the size of the smallest barchan dunes on Mars decreases with increasing atmospheric density over the density range investigated here (~0.01–0.03 kg/m^3^; Fig. 3; Figs. S4–S5). This trend can be described with power laws in terms of dune length, $L\sim {{\rho }_{f}}^{-0.68\pm0.03}$, and dune width, $W\sim {{\rho }_{f}}^{-0.63\pm0.03}$ (${R}^{2}=0.34$ and ${R}^{2}=0.31$, respectively; errors indicate a 95% confidence interval). The observed data scatter around these correlations are both a consequence of local environmental conditions and the automatic nature of the measurements. For example, dune width—a measurement more sensitive to illumination—displays greater scatter than dune length near the pole. A comparison of atmospheric densities obtained using the hypsometric equation (Equation M3; “Methods”) and from a global circulation model during seasons of active sand transport revealed that seasonal variations in atmospheric density do not have a significant impact on these results (Supplementary Information; Fig. S7).

## Discussion

First surveys of barchan dunes on Mars, prior to the Mars Reconnaissance Orbiter^18,30^, were limited by image resolution and reported average dune sizes of ~500 m^18,30^. Here, we find that small barchan dunes dominate the dataset, and consequently report much smaller values of average dune sizes: $220{{{{{\rm{m}}}}}}$ at mid ($70^\circ {{{{{\rm{S}}}}}}-70^\circ {{{{{\rm{N}}}}}}$) and $160$ m at high (>$70^\circ {{{{{\rm{N}}}}}}$, Table 1) latitudes. The large number of small dunes at high northern latitude decreases the average dune size near the pole relative to lower-latitude dunes (70°S–70°N). The pixel size of the Context Camera (CTX) mosaic we employ (5–6 m/pixel) is much smaller than the typical size of dunes, precluding the possibility that dunes were not detected due to insufficient spatial resolution.

**Table 1:** Summary of barchan morphometrics on Mars and comparison with Earth (rounded to nearest integer; P_2_ and P_98_ denote the 2nd and 98th percentiles, respectively)

	Width, *W* (m)			Length, *L* (m)			Height, *H* (m)		
	Mean	P_2nd_	P_98th_	Mean	P_2nd_	P_98th_	Mean	P_2nd_	P_98th_
Mars* (>70°N) *N* = 627,006	160	79	365	150	81	343	21	9	48
Mars (70°S-70°N), *N* = 19,128	212	84	471	214	83	485	26	9	61
Earth (Supplemental Information) *N* = 2931	95	16	316	67	14	226	12	2	51

^*^Barchans in the northern polar sand sea constitute ~97% of our dataset. See Tables S1–S2 for validation and S3 for compilation of terrestrial data.

The size and shape of barchan dunes are controlled by a complex interplay between both the global properties of the atmosphere and local perturbations, e.g., from topography, dune-dune interactions, local variations in excess wind shear velocities, or near-surface volatiles^24,29,31,32^ (Supplementary Information). Such perturbations lead to the observed scatter in dune dimension for any given atmospheric density, whereas the first-order control of atmospheric properties is reflected by the global relationship between dune dimensions and atmospheric density when local effects are averaged (Fig. 3). While the higher relative abundance of smaller dunes at high northern latitudes is likely due to the lower elevation, and thus, higher atmospheric pressure throughout Mars’ northern lowlands, the greater scatter in dune size in that region could potentially arise from cryogenic processes, which were found to affect the saltation saturation length scale and, as a result, dune growth (Fig. S9)^33,34^.

Importantly, both the wavelength of large dark-toned ripples^35,36^ and length of the smallest barchan dunes decrease with increasing atmospheric density, robustly and tightly bounding the wavelength gap that would arise from hydrodynamic theory (Fig. 3; Figs. S6–S8). The best-fit power law between dune size and atmospheric density has an exponent close to −2/3—the same value as that empirically derived for subaqueous ripples^1,5,11^. In addition, the departure from a true inverse relationship, as would be expected if dune size was solely controlled by transport saturation length^1,5,11^, confirms that the viscous length scale $\left(\frac{\nu }{{u}_{*}}\right)$ impacts the initial size of martian dunes as well, lending quantitative support to the hydrodynamic model of ref. 11. Thus, these observations are readily consistent with a hydrodynamic origin of large martian ripples^1,5,6,12,37^. Under this hypothesis, the smallest bedforms arising from that hydrodynamic anomaly on Mars—meter-scale ripples—are about an order of magnitude smaller than terrestrial protodunes.

Like larger aeolian dunes, these meter-scale ripples may grow through time, but their growth would be limited by the hydrodynamic anomaly arising from Mars’ low-density atmosphere (which increases the atmosphere’s kinematic viscosity, explaining their analogy to subaqueous ripples), triggering erosion at the crest of bedforms with wavelengths of a few meters to several decameters. Whereas their size is not the most favored on Mars (i.e., their growth rate is not highest), ~80 m-scale incipient dunes may also form (and subsequently grow) because the formation of smaller dunes that could prevent their inception is precluded by the hydrodynamic anomaly^12^. Specifically, the observed decrease in large-ripple and dune sizes with increasing atmospheric density is quantitatively consistent with the existence of a hydrodynamic anomaly defined by a constant range in bedform-scale Reynolds number, ${{{{{{\rm{Re}}}}}}}_{ \lambda }=\frac{ \lambda {u}_{*}}{\nu } \sim 5\times {10}^{3}-6\times {10}^{4}$ (where ${u}_{*}$ is the wind shear velocity; “Methods”) under martian surface conditions. This remarkable agreement between model predictions and observations on Mars’ surface strongly supports the hypothesis that large martian ripples are more analogous to drag ripples and aeolian dunes than to aeolian impact ripples^1,5,12^. Thus, ancient aeolian sandstones may disclose the density of Mars’ atmosphere at the time of sediment deposition through reconstructions of windblown bedform sizes^1,38^, offering prime exploration targets to unravel the evolution of Mars’ early atmosphere and surface environments.

## Methods

### Detection of barchan dunes on Mars with machine learning

Our global database of barchan dune morphometrics on Mars was compiled using a convolutional neural network (CNN), providing the fullest description of martian barchan dunes to date. Data were processed with minimal human intervention. Whereas manually processed data typically present higher accuracy per sample, the errors in the average morphometrics reported here and that were potentially introduced by our automatic analysis are mitigated in a central-limit theorem sense. In addition, the global coverage of our data provides an outlook into otherwise elusive spatial trends in dune morphology.

Dune fields on Mars were globally cataloged and classified manually in prior studies^20,22^, and the morphology of individual barchan dunes was studied locally^18,29^. These studies did not generate datasets sufficient in size in which the smallest dunes could be identified in a statistically robust way. Such a survey of the smallest dunes on Mars requires an exhaustive sample of dunes. However, compiling such an extensive dataset has been challenging to date due to the large number of dunes on Mars and the time-consuming nature of manual measurements. Barchan dunes are ideal objects for machine-learning assisted detection because they appear as isolated units and their unique crescentic shape makes them more readily detectable by both humans and machines compared to other types of dunes^26^.

Traditional computer vision techniques rely on sharp image gradients for object detection and are inaccurate when the objects and their environment have similar colors or textures^39^. Recently, deep machine learning algorithms have revolutionized object detection in images, achieving human-like abstraction^40^. These CNNs excel at supervised learning tasks such as finding a set of parameters that, given a set of inputs, best reproduces a set of expected outputs. To extract dune contours on Mars, we used Mask R-CNN, a state-of-the-art supervised instance segmentation CNN^41^. We first manually labeled isolated barchan dunes in a set of images extracted from the global MRO CTX (5–6 m/pixel^42^) mosaic^43^. We elected to exclude connected barchanoidal ridges that are more difficult to analyze automatically. Using this dataset, we optimized the parameters of Mask R-CNN through training, until its accuracy (the mean average precision evaluated using a separate test dataset^26^) reached 77%. Our dataset included 1074 images of barchan dunes, uniformly selected across the martian surface to obtain a well balanced dataset. The training and validation datasets were composed of 80 and 20% of the total manually labeled data, respectively. Mean average precision (mAP) was determined from an additional test dataset composed of 50 images. To avoid overfitting, we employed image augmentations (rotations, image saturation, contrast), as well as L2 regularization. The full set of hyperparameters of the trained model can be found in ref. 26. While training the model further would have likely resulted in higher accuracy and better outlier detection, we found this mAP value sufficient for our goal of characterizing the first-order morphometrics of barchan dunes on Mars. The trained model was then used to detect isolated barchans in the global CTX mosaic^43^. A full discussion of training process and model evaluation is provided in ref. 26, and a discussion of the errors associated with our statistical analysis is provided in the Supplementary Information.

### Extraction of dune morphometrics from dune outlines

Of the 137,111 CTX images extracted automatically from the global CTX mosaic and examined by the CNN, 55,674 were found to contain at least one instance of an object identified as an isolated barchan dune. To measure the morphometrics of each identified dune, we automatically identified six reference points along the dune contour—the slipface center, the horns’ apexes, the tail, and the dune sides^28^ (Fig. S1). The slipface center (middle of the base of the slipface) was identified as the deepest convexity defect along the dune contour. Horn apexes were mapped as the two intersection points between the dune contour and its convex hull closest to the slipface center. The tail of the dune was set as the point furthest away from the slipface along the dune’s stoss (Supplemental Material). Finally, the sides of the dunes were detected as the two extreme points along the dune contour in the direction perpendicular to a vector drawn between the tail and the slipface center (Fig. 1 and Fig. S1). The dune length was measured as the distance between the tail and the slipface center, the dune width as the distance between its sides, and the horn lengths (not discussed in this work) as the distances between the horn apexes and a line passing through the slipface center, normal to the tail-slipface vector. We estimated dune height by multiplying the horizontal length of the slipface (measured from the brink to the base of the slipface, in map view) by the tangent of 30° as a representative angle of repose for martian aeolian sand^6^, and dune volume, as the product of *H*/2 (as a proxy for the average height of all points along the dune’s surface) and basal area within the dune outline (Fig. S1).

### Data filtering

To increase the robustness of our compilation, we filtered our dataset as follows:

(1) Between 70°S–70°N, we only used images of areas previously mapped as dune fields^20,22^. This was done to save computation time – since our goal is not to detect new barchan dunes on Mars but to characterize the morphology of dunes on Mars on a global scale.

(2) Because barchans tend to occur in fields rather than as solitary landforms, we discarded images containing less than three objects to remove potentially spurious detections.

(3) Upon manually inspecting our results, we found that the model misclassified some dark sublimation-driven features at southern polar latitudes, such as Dalmatian spots and spiders^44^, as barchans. Consequently, we elected to discard dunes in latitudes poleward of 70°S.

(4) By removing dunes with detection-confidence levels outputted by the detection algorithm lower than 70%, and dunes with convexity defects smaller than 2.5% of total dune length (*L* + mean horn length). The latter step removed many isolated objects erroneously identified as dunes, but also isolated dome dunes, which are excluded from this study. Our choice of 70% confidence is based on trial and error and visual inspection of the detected objects.

Upon filtering, our final dataset contained 646,134 dunes (covering an area of about $15\times {10}^{3}\,{{{{{{\rm{km}}}}}}}^{2}$), roughly 97% of which are located poleward of 70°N. We use orthogonal distance regression^45^ to describe various correlations between dune morphometrics as best-fit power laws (Fig. 2b–g). To filter for intra-crater dunes south of 70°N, we used craters locations and diameters as mapped manually^46^. We find that south of 70°N, 3,819 dunes in our database are located in the inter-crater terrain, and 15,309 dunes are found within craters.

### Validation of automatically computed morphometrics

The quality of our final dataset was validated manually by comparing automatically measured morphometrics to manually measured morphometrics in CTX images using JMARS^47^. Additionally, the extracted morphometrics data was validated with a smaller test dataset of manually measured barchan morphometrics on Mars^48^ (Figs. S3–S4) as well as with individual HiRISE digital terrain models (Supplementary Information; Fig. S5).

### Width of the hydrodynamic anomaly on Mars

Under martian surface conditions, the gap in bedform sizes arising from the Hanratty anomaly was predicted to correspond to ${{{{{{\rm{Re}}}}}}}_{ \lambda } \sim 5\times {10}^{3}-6\times {10}^{4}$^12^. We expressed the width of the hydrodynamic anomaly in terms of bedform wavelength, $\lambda$, as

1 $$\lambda=\frac{\nu }{{u}_{\ast }}{{{{{{\rm{Re}}}}}}}_{ \lambda },$$

where ${{{{{{\rm{Re}}}}}}}_{ \lambda } \sim 5\times {10}^{3}-6\times {10}^{4}$ and wind shear velocity, ${u}_{*}$, was estimated to a first order as the transport fluid threshold, ${u}_{*{{{{{\rm{f}}}}}}}$,

2 $${u}_{\ast {{{{{\rm{f}}}}}}}=\sqrt{\frac{{\rho }_{s}-{\rho }_{f}}{{\rho }_{f}}gd\theta },$$

where ${\rho }_{s}\,\approx\, 2900$ kg/m^3^ is the density of basaltic sediment grains, $g\,\approx\, 3.71$ m/s^2^ is the surface acceleration of gravity, $d\,\approx\, 125$ μm is sediment grain diameter^7^ and $\theta=0.01$ is the critical Shields number as constrained from Mars-like low-pressure wind tunnel experiments^49^. We tested the sensitivity of the derived bedform-gap width to the chosen threshold model by comparing this formulation with additional fluid threshold models^16,50^, and found this choice does not affect our conclusions (Figs. S6–S8). Because dune-forming winds ought to be larger than the impact threshold for sand transport (${u}_{*{{{{{\rm{i}}}}}}}$), we also calculated the width of the Hanratty gap by equating ${u}_{*}$ to values derived from impact-threshold models^25,51,52^ (not shown) and found a similar overlap between predicted bedform gaps and the observed range of missing bedform wavelengths on Mars. We note that these results could be used to constrain formative wind shear velocities within a given dune field from the observed range of missing bedform wavelengths.

### Estimation of atmospheric density from topography

We computed the surface atmospheric density, ${\rho }_{f}$, on Mars assuming an isothermal ideal-gas CO_2_ atmosphere (${m}_{{{{{{\rm{c}}}}}}{{{{{{\rm{o}}}}}}}_{2}}=44.01{{{{{\rm{g}}}}}}/{{{{{\rm{mol}}}}}}$) with surface pressure of ${P}_{0}=610{{{{{\rm{Pa}}}}}}$, temperature ${T}_{0}=230{{{{{\rm{K}}}}}}$ and a scale height of *H* = 12 km^53^,

3 $${\rho }_{f}={\rho }_{0}\exp (-\frac{z}{H})$$

where ${\rho }_{0}=\left({P}_{0}{m}_{{{{{{{\rm{co}}}}}}}_{2}}\right)/\left(R{T}_{0}\right)$, $R=8.3145{{{{{\rm{J}}}}}}{{{{{{\rm{mol}}}}}}}^{-1}\,{{{{{\rm{K}}}}}}$ is the ideal gas constant, and the height, $z$, was measured using Mars Orbiter Laser Altimeter data. To produce Fig. 3, we binned atmospheric density and dune sizes between latitudes -$70^\circ {{{{{\rm{N}}}}}}$ and $90^\circ {{{{{\rm{N}}}}}}$ in equal-area spatial bins of 60 km^2^ (1° near the equator) and plotted the 2nd percentile of dunes length in the bin as a function of its atmospheric density calculated using the mean bin elevation. We confirmed that the statistical properties of the distributions do not vary significantly for bin sizes of 15–60 km^2^. Furthermore, we find that data from dunes located inside impact craters display a slightly higher scatter than dunes outside of craters, a likely results of crater topography on local wind conditions^54^, sediment accumulation, and other environmental factors (Supplementary Information; Figs. S9–S11). However, this increased scatter has a minimal impact on detected trends.

## Supplementary information

Supplementary Information

## Data Availability

The dunes’ morphometrics data analyzed in this study have been deposited in the following repository: 10.6084/m9.figshare.17200205.v1.

## Code availability

Code used to process the data is available here: 10.5281/zenodo.7305150.

## References

[1] Lapôtre MGA (2016). Large wind ripples on Mars: a record of atmospheric evolution. Science.

[2] Baker MM (2018). The Bagnold Dunes in southern summer: active sediment transport on Mars observed by the Curiosity rover. Geophys. Res. Lett..

[3] Tholen K, Pähtz T, Yizhaq H, Katra I, Kroy K (2022). Megaripple mechanics: bimodal transport ingrained in bimodal sands. Nat. Commun..

[4] Lapôtre MGA (2020). Probing space to understand Earth. Nat. Rev. Earth Environ..

[5] Lapôtre MGA, Ewing RC, Lamb MP (2021). An evolving understanding of enigmatic large ripples on Mars. JGR: Planets.

[6] Ewing RC (2017). Sedimentary processes of the Bagnold Dunes: implications for the eolian rock record of Mars. JGR: Planets.

[7] Weitz CM (2018). Sand grain sizes and shapes in eolian bedforms at Gale Crater, Mars. Geophys. Res. Lett..

[8] Gough TR, Hugenholtz CH, Barchyn TE (2021). Re‐evaluation of large Martian ripples in Gale Crater: granulometric evidence for an impact mechanism and terrestrial analogues. JGR Planets.

[9] Sullivan R, Kok JF, Katra I, Yizhaq H (2020). A broad continuum of aeolian impact ripple morphologies on Mars is enabled by low wind dynamic pressures. JGR: Planets.

[10] Lapôtre MGA (2018). Morphologic diversity of Martian ripples: implications for large‐ripple formation. Geophys. Res. Lett..

[11] Lapôtre MGA, Lamb MP, McElroy B (2017). What sets the size of current ripples?. Geology.

[12] Duran Vinent O, Andreotti B, Claudin P, Winter C (2019). A unified model of ripples and dunes in water and planetary environments. Nat. Geosci..

[13] Lü, P. et al. Direct validation of dune instability theory. *Proc. Natl Acad. Sci. USA* **118**, e2024105118 (2021). 10.1073/pnas.2024105118 PMC8092407 33883281

[14] Abrams J, Hanratty TJ (1985). Relaxation effects observed for turbulent flow over a wavy surface. JFM.

[15] Frederick KA, Hanratty TJ (1988). Velocity measurements for a turbulent nonseparated flow over solid waves. Exp. Fluids.

[16] Bagnold, R. A. *The Physics of Blown Sand and Desert Dunes* (W. Morrow & Company, 1942).

[17] Kroy K, Sauermann G, Herrmann HJ (2002). Minimal model for sand dunes. PRL.

[18] Breed CS, Grolier MJ, McCauley JF (1979). Morphology and distribution of common ‘sand’dunes on Mars: Comparison with the Earth. JGR: Solid Earth.

[19] Tsoar H, Greeley R, Peterfreund AR (1979). Mars: The north polar sand sea and related wind patterns. JGR: Solid Earth.

[20] Hayward, R. K. et al. Mars global digital dune database and initial science results. *JGR: Planets*10.1029/2007JE002943 (2007).

[21] Hayward, R. K., Fenton, L. K., Titus, T. N., Colaprete, A. & Christensen, P. R. Mars global digital dune database: MC-30. *USGS OFR* 1259. https://pubs.er.usgs.gov/publication/ofr20101170 (2012).

[22] Fenton LK (2020). Updating the global inventory of dune fields on Mars and identification of many small dune fields. Icarus.

[23] Hersen P, Douady S, Andreotti B (2002). Relevant length scale of barchan dunes. PRL.

[24] Parteli EJR, Duran O, Herrmann HJ (2007). Minimal size of a barchan dune. PRLE.

[25] Claudin P, Andreotti B (2006). A scaling law for aeolian dunes on Mars, Venus, Earth, and for subaqueous ripples. ESSL.

[26] Rubanenko L, Pérez-López S, Schull J, Lapôtre MGA (2021). Automatic detection and segmentation of barchan dunes on Mars and Earth using a convolutional neural network. IEEE JSTARS.

[27] Hesp PA, Hastings K (1998). Width, height, and slope relationships and aerodynamic maintenance of barchans. Geomorphology.

[28] Elbelrhiti, H., Andreotti, B. & Claudin, P. Barchan dune corridors: field characterization and investigation of control parameters. *JGR Earth Surf*. 10.1029/2007JF000767 (2008).

[29] Bourke MC, Goudie AS (2009). Varieties of barchan form in the Namib Desert and on Mars. Aeolian Res..

[30] Lancaster N, Greeley R (1990). Sediment volume in the north polar sand seas of Mars. JGR: Solid Earth.

[31] Zhang, D., Narteau, C. & Rozier, O. Morphodynamics of barchan and transverse dunes using a cellular automaton model. *JGR Earth Surf*. 10.1029/2009JF001620 (2010).

[32] Parteli EJR (2014). Origins of barchan dune asymmetry: insights from numerical simulations. Aeolian Res..

[33] Feldman WC (2008). Hydrogen content of sand dunes within Olympia Undae. Icarus.

[34] Comola F, Gaume J, Kok JF, Lehning M (2019). Cohesion‐induced enhancement of aeolian saltation. Geophys. Res. Lett..

[35] Vaz, D. A., Silvestro, S., Chojnacki, M. & Silva, D. C. Global wavelength survey of Martian bedforms: methods and preliminary results (No. EGU22-6697). Copernicus Meetings. (2022).

[36] Vaz, D. A. et al. Constraining the mode of eolian transport on Mars through a global morphometric survey of bedforms. Supplementary material 10.6084/m9.figshare.21064657.v2 (2022)

[37] Lapôtre MGA, Rampe EB (2018). Curiosity’s investigation of the Bagnold Dunes, Gale crater: Overview of the two‐phase scientific campaign and introduction to the special collection. Geophys. Res. Lett..

[38] Rubin, D. M. et al. Ancient winds, waves, and atmosphere in Gale Crater, Mars, inferred from sedimentary structures and wave modeling. *JGR: Planets* **127**, e2021JE007162 (2022).

[39] Forsyth DA, Ponce J (2003). A modern approach. Comput. Vis. Mod. Approach.

[40] Goodfellow, I., Bengio, Y. & Courville, A. *Deep Learning* (MIT Press, 2016).

[41] He, K., Gkioxari, G., Dollár, P. & Girshick, R. Mask R-CNN. *IEEE International Conference on Computer Vision (ICCV)*, 2017, pp. 2980–2988, 10.1109/ICCV.2017.322.

[42] Malin, M. C. et al. Context camera investigation on board the Mars Reconnaissance Orbiter. *JGR: Planets*10.1029/2006JE002808 (2007).

[43] Dickson, J. L., Kerber, L. A., Fassett, C. I. & Ehlmann, B. L. A global, blended CTX mosaic of Mars with vectorized seam mapping: A new mosaicking pipeline using principles of non‐destructive image editing. In *LPSC* Vol. 49, 1–2 (2018).

[44] Zuber MT (2003). Learning to think like martians. Science.

[45] Markovsky I, Van Huffel S (2007). Overview of total least-squares methods. Signal Process..

[46] Robbins, S. J. & Hynek, B. M. A new global database of Mars impact craters ≥ 1 km: 1. Database creation, properties, and parameters. *JGR: Planets*10.1029/2011JE003966 (2012).

[47] Christensen, P. R. et al. JMARS-A planetary GIS. American Geophysical Union (Fall). In *Abstracts* Vol. 1, 6 (2009).

[48] Sherman, D. J., Zhang, P., Bae, J., Butler, R. & Rew, H. Barchan dunes: Geomorphometric data 10.5281/zenodo.5637339 (2021).

[49] Andreotti, B., Claudin, P., Iversen, J. J., Merrison, J. P. & Rasmussen, K. R. A lower-than-expected saltation threshold at Martian pressure and below. *Proc. Natl Acad. Sci. USA* **118**, e2012386118 (2021). 10.1073/pnas.2012386118 PMC7865126 33509927

[50] Swann C, Sherman DJ, Ewing RC (2020). Experimentally derived thresholds for windblown sand on Mars. Geophys. Res. Lett..

[51] Pähtz T, Kok JF, Herrmann HJ (2012). The apparent roughness of a sand surface blown by wind from an analytical model of saltation. N. J. Phys..

[52] Kok, J. F. An improved parameterization of wind‐blown sand flux on Mars that includes the effect of hysteresis. *Geophys. Res. Lett*. 10.1029/2010GL043646 (2010).

[53] Forget, F. et al. Density and temperatures of the upper Martian atmosphere measured by stellar occultations with Mars Express SPICAM. *JGR: Planets*10.1029/2008JE003086 (2009).

[54] Gunn, A., Rubanenko, L. & Lapôtre, M. G. A. Accumulation of windblown sand in impact craters on Mars. *Geology*10.1130/G49936.1 (2022).

