# Supplementary material for: A distinct ripple-formation regime on Mars revealed by the morphometrics of barchan dunes
Source: Nat Commun. 2022 Nov 22;13:7156. doi: 10.1038/s41467-022-34974-3 (PMC9684498; doi:10.1038/s41467-022-34974-3)
Supplement: Supplementary file 1 — Supplementary Information [file 41467_2022_34974_MOESM1_ESM.pdf]

Supplementary Information for "A distinct ripple-formation regime on Mars revealed by the morphometrics of barchan dunes"

Lior Rubanenko[1], Mathieu G.A. Lapôtre[1], Ryan C. Ewing[2], Lori K. Fenton[3], Andrew Gunn[1,4].

[1]Department of Geological Sciences, Stanford University, Stanford, CA 94305, USA.
[2]Department of Geology & Geophysics, Texas A&M, College Station, TX 77843, USA.
[3]Carl Sagan Center, SETI Institute, Mountain View, CA 94043, USA.
[4]School of Earth, Atmosphere & Environment, Monash University, Clayton, VIC 3800, Australia.

Measurements of barchan morphometrics from dune contours

After extracting the outlines of dunes using machine learning [1], dunes morphometrics were measured by identifying six reference points along their outlines – the center of the slipface, the apexes of horns, the tail (edge of the dune in the upwind direction) and the sides. To measure the morphometrics of dunes, we developed the following pipeline:

(a) The dune slipface center is found as the deepest convexity defect of the contour (Figure S1a,b).
(b) The apexes of the horns are identified as the intersection points between the convex hull of the dune and the contour, closest to the slipface (red outline in Figure S1c,d).
(c) After identification of the slipface center and horn apexes, the dune is rotated such that its migration direction faces "up". When the dune is symmetric (i.e., with long-to-short horn lengths ratio < 1.5), the migration direction is estimated as the bisector to the vectors connecting the slipface center to the horns' apexes. When the dune is asymmetric (i.e., with long-to-short horn lengths ratio > 1.5), its migration direction is approximated as the direction of the vector connecting the dune tail to the slipface center. The tail of the dune is identified as the point furthest away from the horns in the lower (downwind) half of the dune. The dune sides are calculated as the two extreme points in the direction perpendicular to the migration direction.
(d) The height of the dune is measured automatically from the planview-projected length of the slipface (Figure S1e,f).
    (i) To measure the planview length of the slipface, we first automatically extract a profile of the pixel intensity of the CTX image along a line passing through the slipface center and perpendicular to the dune contour.
    (ii) The profile is smoothed using a gaussian filter with window size of 2 pixel and calculate its absolute value (to only have positive smoothed grayscale values along the profile).
    (iii) We use a peak detection algorithm to find the width of the most prominent extremum with respect to the median brightness of pixels around it (Figure S1e).

(iv) The error in our method of measurement is at least 5 meters, the resolution of a CTX pixel. However, effects such as sand aprons and illumination may exacerbate the error – especially in high latitudes. See Figure S9.

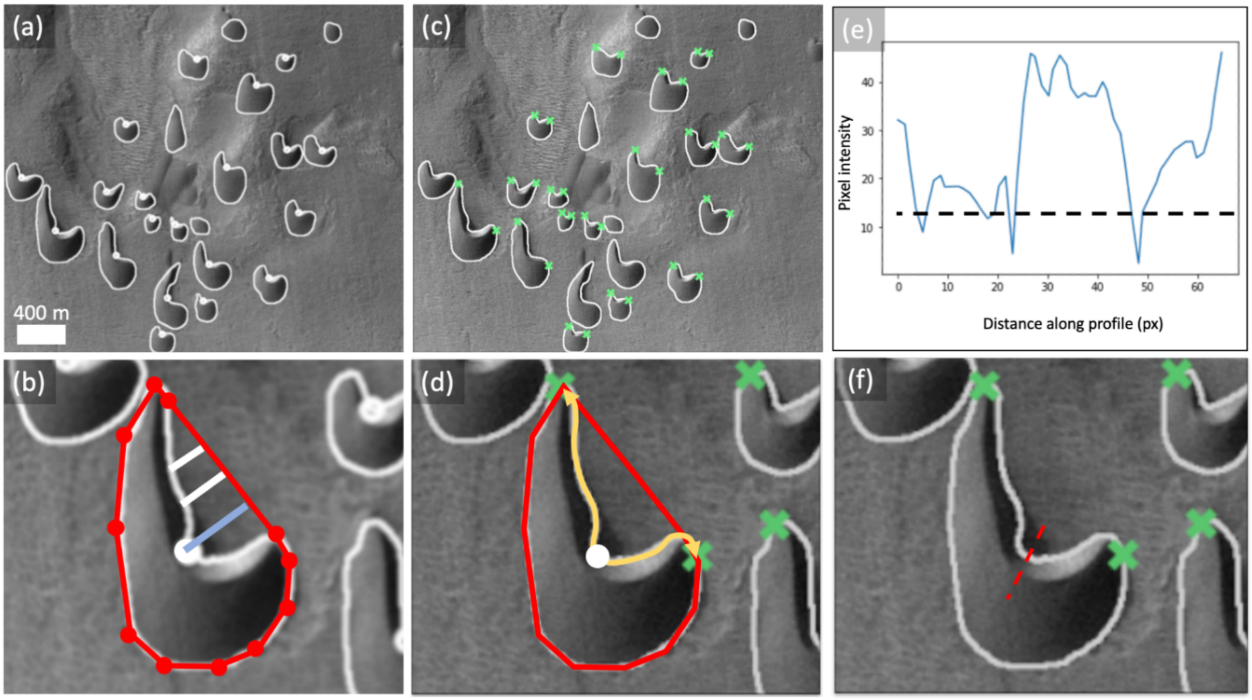

Supplementary Figure S1: (a,b) Detecting dune slipfaces as the deepest convexity defect. Red contour and points shows the convex hull of the contour (c,d) Detecting the horns' apexes as the intersection points between the contour and its convex hull. Yellow arrows are for demonstrating the algorithm iteration along the dune contour (e,f) Inferring dune height from the planview length of the slipface (in map view). The width of the slipface is calculated as the width of the most prominent peak in pixel brightness along a profile passing through the slipface center perpendicular to the dune contour. Image derived from the global CTX catalog (-42.20°N, -31.89°E).

Uncertainty of derived scaling laws and mitigation

A full discussion of potential errors of our machine learning approach was previously published in an analysis of detection performance [1]. To compute the orthogonal distance regression parameters corresponding to the regression in Figure 2, we employed the Python Scipy ODR class, which is based on the ODRPACK Fortran implementation of the Levenberg-Marquardt algorithm [2]. As an estimate of the error on our scaling law parameters derived using orthogonal regression, we use the standard deviation of the fitted parameters (slope and intercept in log-log scale; see discussion in [3]). For our orthogonal regression lines, we calculate $R^2 = 1 - RSS/TSS$, where RSS and TSS are the residual and total sum of squares. For the log-transformed relationship in Figure 3 and Supplementary Figures we use linear regression (assuming density is not a response variable, as it is measured from topography or by the GCM). We employ the

Python Scipy linregress function and estimate the errors as the standard errors of the fitted parameters.

Additional examples of dunes detected using the machine learning model

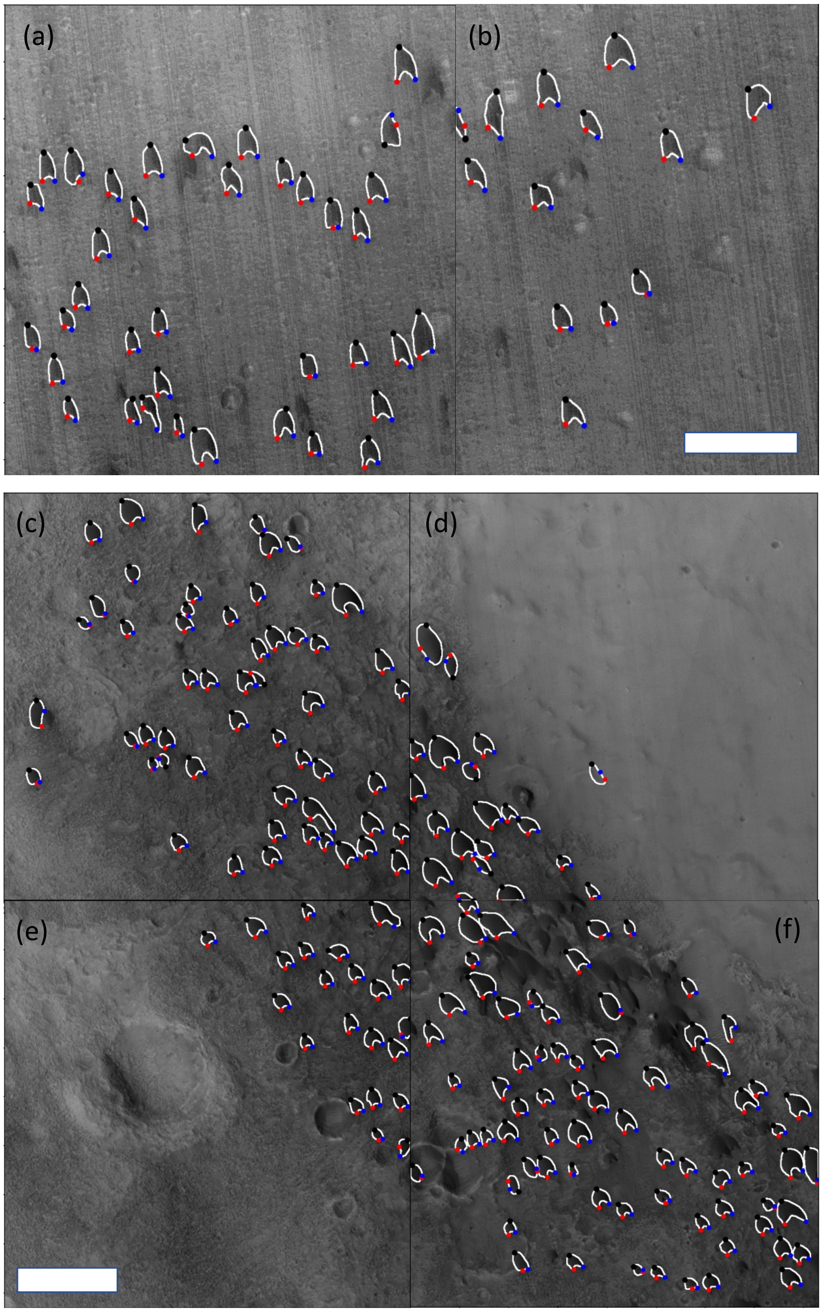

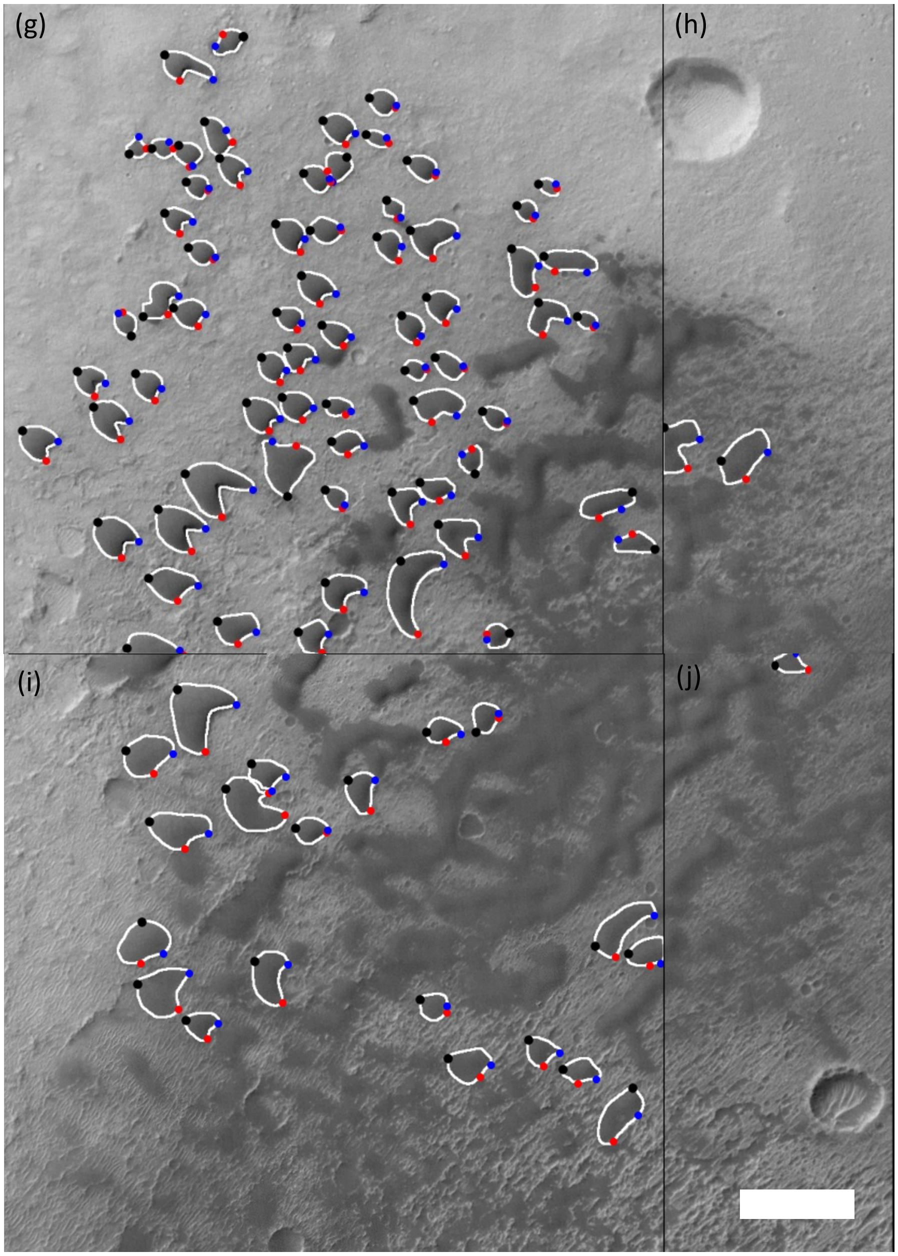

Supplementary Figure S2: Machine-learning-detected dunes in (a-b) Oyama Crater (23.23°N, -20.4°E) (c-f) 36.66°N, -0.76°E and (g-k) -31.25°N, 152.703°E. Red, blue, and black dots indicate the automatically detected dune horns (left and right) and tail, respectively (Figure S1). North is up. Each panel describes a 800x800 pixel segment cropped out of the global CTX mosaic [4], as described in the Methods. Data was subsequently filtered as described in the main text to remove spurious detections. Scale bars represent 1 km. North is up in all images.

Validation of morphometric database with manually measurements

To validate individual dune measurements, we compare manual measurements made in JMARS to automatic measurements made using our algorithm for 10 random dune fields on Mars. Figure S3 illustrates one of these comparisons for the dune field shown in Figure 2 (-42.20°N, -31.89°E).

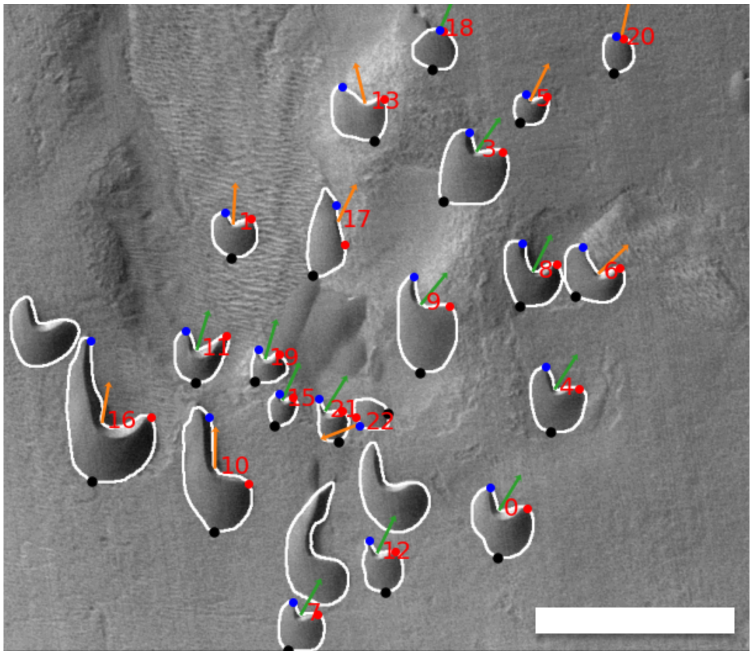

Supplementary Figure S3: A dune field used for manual validation (-42.20°N, -31.89°E). Supplementary Tables S1–S2 report the width and length measurements for each indexed dune in the image. Scale bar represents 1 km. North is up.

Index	Manual Measurement on CTX image (m)	Automatic Measurement (m)	error
0	272	260	4%
1	181	180	<1%
2	-	-	-
3	320	327	2%
4	234	235	<1%
5	140	134	4%
6	178	180	<1%
7	163	202	20%
8	211	199	6%
9	350	370	6%
10	355	345	3%
11	243	250	3%
12	217	220	1%
13	233	211	10%
14	-	-	-
15	156	147	6%
16	335	324	3%
17	-	-	-
18	-	-	-
19	135	134	<1%
20	-	-	-
21	190	183	1%
22	-	-	-

Table S1. Manual validation of dune length measurements (rounded to nearest integer).

Index	Manual Measurement on CTX image (m)	Automatic Measurement (m)	% error
0	366	%	5%
1	248	251	<1%
2	-	-	-
3	354	390	10%
4	344	354	3%
5	180	187	<1%
6	345	371	7%
7	290	281	3%
8	378	369	2%
9	460	461	<1%
10	320	380	16%
11	258	295	13%

12	253	273	7%
13	271	310	13%
14	-	-	-
15	174	158	10%
16	542	530	2%
17	-	-	-
18	-	-	-
19	220	204	7%
20	-	-	-
21	220	236	6%
22	-	-	-

Table S2. Manual validation of dune width measurements (rounded to the nearest integer).

Automatically detected morphometrics are further compared with manual measurements of barchan dunes on Mars (Sherman et al., 2021). The best-fit power law describing the planview dimensions of automatically detected barchans is close to that describing the planview dimensions of manually detected barchans (blue and orange lines in Figure S4).

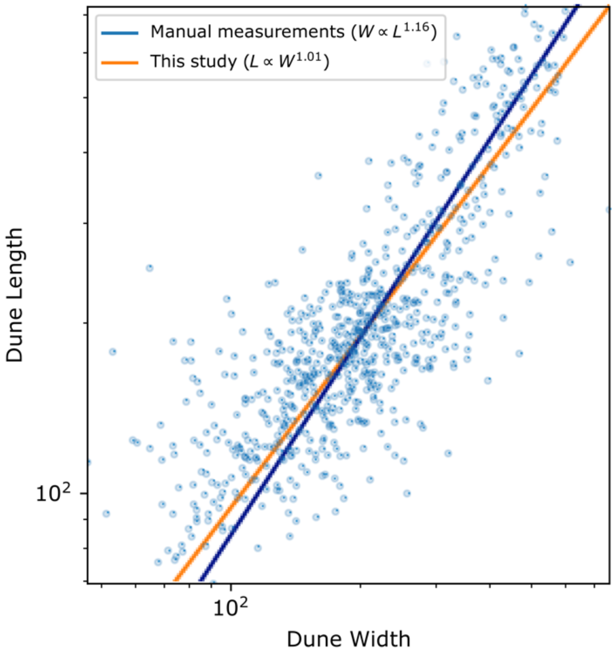

Supplementary Figure S4: Comparison between automatically (this study) and manually (Sherman et al., 2021) outlined and analyzed data for martian barchan dunes.

Validation of dune-height measurements with HiRISE stereographic DTM

The correlation between barchan width and height is weaker than between length and height (see R^2 values in Figure 2), which may partially result from the higher uncertainty associated with measuring width perpendicular to dune migration direction. In addition, our assumptions that the dune brink is the highest part of the dune and that the slipface is close to the angle of repose may not always be accurate, potentially increasing data scatter.

We validate our height-determination algorithm by manually measuring the heights of 22 barchan dunes in Oyama Crater using a HiRISE digital elevation model [5] (image ESP_042714_2035) of a dune field centered around 23.23°N, -20.4°E. Vertical precision is < 1 m (Figure S5).

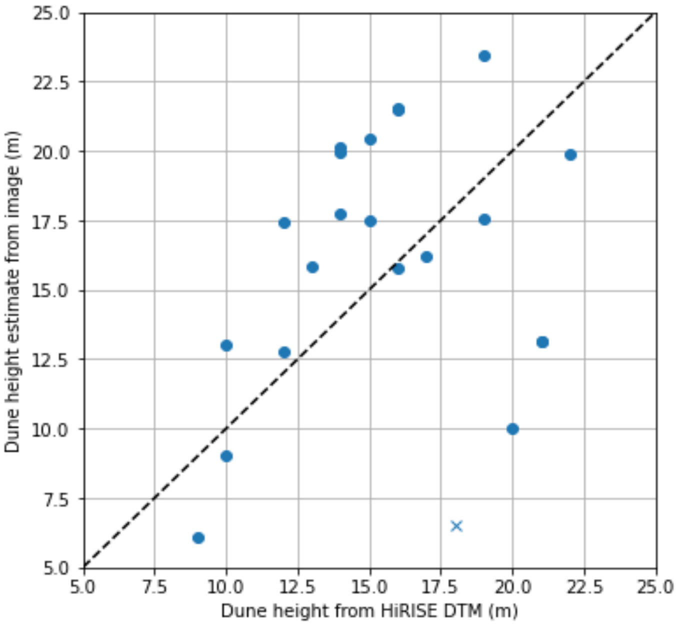

Supplementary Figure S5. Comparison between automatically estimated dune heights and dune heights as manually measured from a HiRISE digital elevation model (ESP_042714_2035). Dashed line highlights a 1:1 relationship. The 'x' symbol (bottom right corner) denotes a dune whose contours were not adequately detected by the machine learning algorithm.

Compilation of terrestrial barchan dune morphometrics

Our morphometric dataset of terrestrial barchan dunes (Figure 2) was compiled from previously published studies with attention to providing a diversity of dune-field settings and geography (Table S3). The full dataset can be accessed through at https://doi.org/10.6084/m9.figshare.17200205.v1.

Location	Citation	Comment
Egypt (N = 111)	[6]	Length was measured as the length of the windward side
China (N = 71)	[7]	
Saudi Arabia (N = 22)	[8]	Length was derived by adding the slipface and the stoss lengths
Morocco (N = 8)	[9]	
Peru (N = 45)	[10]	Length was measured from the tail to slipface
Brazil (N = 47)	[11]	Length was measured as the length of the windward side
California (N = 42)	[12,13]	In [10], length was measured as the length of the stoss side
Kuwait (N = 10)	[14]	Length was derived by adding the slipface and the stoss lengths
Multiple locations, including Algeria, Yemen, Saudi Arabia, Western Sahara, Chad, Peru, China, Namibia, Sudan, Iran, and Niger (N = 2686)	[15]	Length was measured from the tail to slipface

Table S3. Locations and references for compiled terrestrial barchan-dune morphometrics.

Correlation between atmospheric density and dune width

To further validate the result presented in Figure 3, we additionally compute the power law relationship between dune width and atmospheric density. As expected, since the relationship between width and length is close to linear, the power law exponent relating dune width and atmospheric density is, too, close to -2/3 (Figure S6).

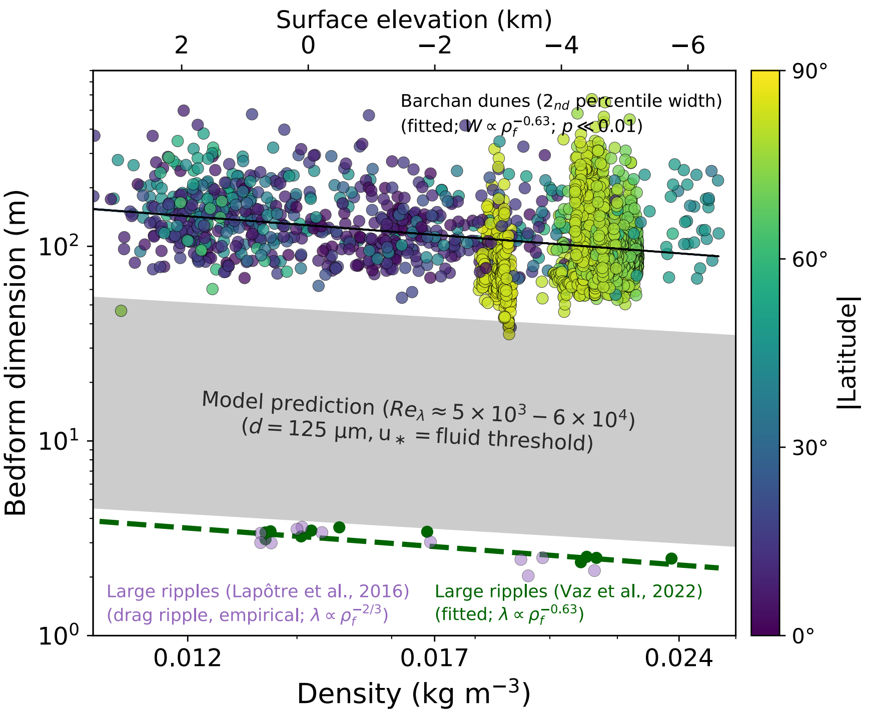

Supplementary Figure S6: Decrease in dune width (blue-to-yellow circles) and large-ripple wavelength (light purple [16] and dark green [17] circles) with increasing atmospheric density. Ripple data follows a previously derived scaling relationships for drag ripples [18]; best-fit power law to an updated global compilation of large-ripple wavelength has an exponent of -0.63 (green dashed line) [17]. The width of the hydrodynamic anomaly (gray shade) was calculated for a 125-µm grain size [19] and assuming that wind shear velocity is at the transport threshold [20] (Methods). The statistical significance of the regression slope was computed using a Wald Test, and margins (smaller than line width(indicate a 95% confidence interval.

Sensitivity of the derived bedform gap to transport threshold model

To test whether the width of the bedform gap we calculate for Mars is affected by the chosen transport threshold model, we tested three different formulations derived from theoretical and empirical considerations. We find that the bedform gap is bounded by the empirically derived scaling laws predicting ripple wavelength and dune length as a function of atmospheric density for all tested models; thus, our conclusions do not depend on the choice of the threshold model (Figure S7). We also calculated the width of the bedform gap equating wind shear velocity to several impact threshold models [21–23]. We find that the predicted gaps overlap with some observed dune data for models that predict stronger transport hysteresis $\left(\frac{u_{*\mathrm{i}}}{u_{*\mathrm{f}}} < \sim 0.3\right)$, consistent with the fact that true formative shear velocities ought to be larger than the impact threshold for sand transport. We note that the model proposed by Andreotti et al. (2021) [20] fits dunes and ripples dimensions slightly better than the other two models we employed; our measurements could potentially be employed to calibrate threshold models.

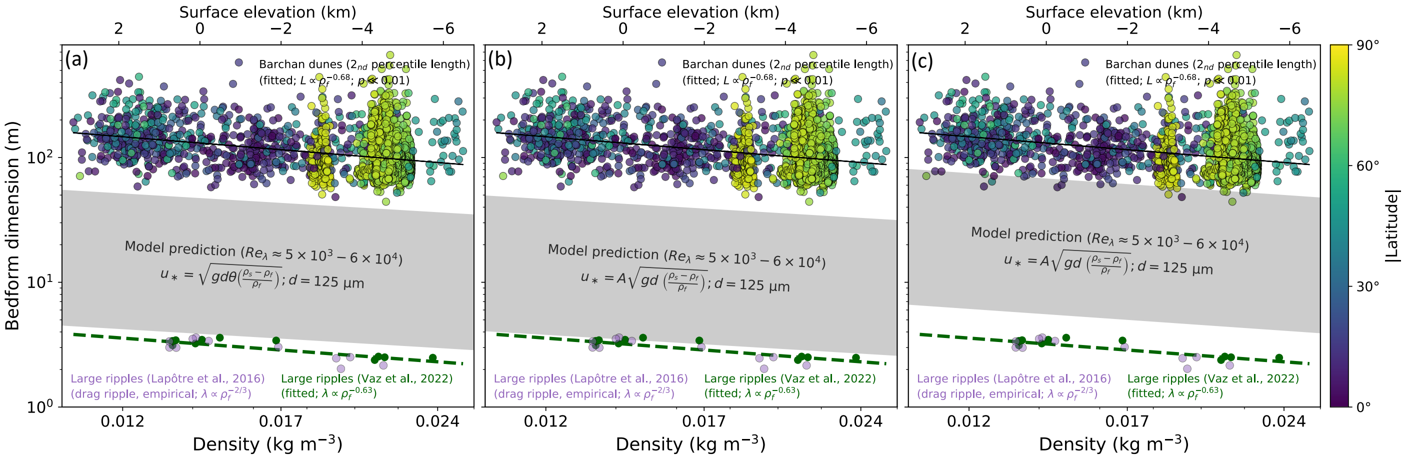

Supplementary Figure S7: Decrease in dune length with atmospheric density, for different fluid threshold models. (a) Andreotti et al. (2021) [20]: $\theta = 0.01$ is the critical Shields number (b) Bagnold (1942) [24]: $A = 0.11$. (c) Swann et al. (2020) [25]: $A = 0.0646\, d_*^{0.2426}$, where $d_* = d\left[\rho_f g \frac{(\rho_s-\rho_f)}{\mu^2}\right]^{\frac{1}{3}}$ and $\mu = 10.8\times10^{-6}$ Pa s. In all panels, $g = 3.71\ \mathrm{m/s^2}$ is the graviational acceleration on Mars, $\rho_s = 2900\ \mathrm{kg/m^3}$ is sediment density, ρ_f is atmospheric density and $d = 125$ µm is grain diameter.

Seasonal variations in atmospheric density

To test if the trends we detect are sensitive to seasonal variations in density, we compared results obtained using the hypsometric equation (Equation M3) with outputs from a global climate model (GCM) [26]. Because we are only interested in the value of atmospheric density at times of active sand transport, we calculate the average annual atmospheric density, weighted by the magnitude of sand flux as calculated from GCM outputs, as a characteristic value of atmospheric density during the dune-forming season. We use the GCM to extract atmospheric density and wind stress globally on Mars in intervals of 10° solar longitude, and estimate the sand flux using the formulation of [27], $q = C_q \frac{u_{*,it}}{g}\rho_f(u_*^2 - u_{*\mathrm{t}}^2)$, where $C_q = 6.1$, $u_{*\mathrm{t}}$ is the threshold wind shear velocity (estimates using the value used in [28] as is appropriate for GCM modeling), u_* is the surface wind shear velocity, ρ_f is the density and $g = 3.71\ \mathrm{m\,s^{-2}}$ is the acceleration due to gravity. We verified that our result does not significantly change when other flux laws are employed [29].

The observed trend between dune size and atmospheric density does not significantly change when density is computed using the GCM (Figure S8). The GCM predicts a slightly higher atmospheric density in latitudes > 70°N relative to when assuming an isothermal atmosphere (Figures 1 and S6–S7), which results in a slightly lower fitted power-law exponent. When only fitting data in latitudes < 70°N, we again obtain an exponent that is close to the theoretically predicted -2/3. We note that scatter in dune size is greater near the pole, implying other mechanisms may be involved in controlling dune size there, as discussed in the next section (Figure S9). Furthermore, GCM predictions are likely to be less accurate near the poles, where significant filtering is applied due to the nature of traditional latitude/longitude GCM grids with converging meridians; as a result, the GCM may fail to capture local phenomena such as slope or thermal-contrast winds near the polar cap.

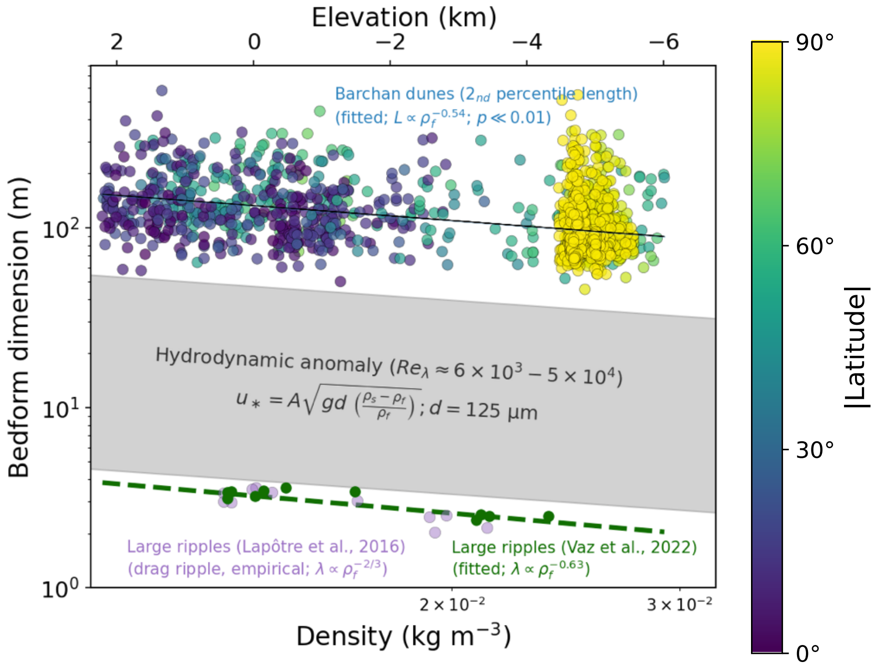

Supplementary Figure S8: Decrease in dune length with mean atmospheric density weighted by sand flux magnitude, computed using a global climate model. The slightly higher mean density in the polar region of Mars decreases the exponent of the fitted power law. However, when removing data > 70°N, we obtain a similar power-law exponent as in Figure 3 (-0.58). The resolution of the data is affected by the GCM resolution near the pole.

Potential impact of near-surface volatiles at high latitudes

Overall, Mars' smallest barchan dunes (2nd percentile, Table 1) have widths and lengths of ~80 m, *i.e.*, they are ~5–6 times larger than their terrestrial counterpart. Dune size and shape vary between equatorial and polar regions, which captures an 11 km latitudinal average elevation range and a three-fold increase in density. In the north polar region (>70°N), which has the

greatest abundance of dunes at the lowest elevations, barchan dunes are on average smaller than barchan dunes at lower latitudes (70°S-70°N; Table 1) and are more elongated (see discussion in [30]), with the longest and narrowest dunes concentrated along the sand sea's northernmost margin (Figure S9). Compared with lower-latitude dunes, which are mainly located within impact craters (Figure S10), north-polar barchans have a more contiguous distribution, providing an opportunity to investigate spatial patterns in sizes and shapes. Much like length-to-width ratios, dune volumes vary dramatically across the north pole (Figure S9). In Olympia Undae, barchans are 2–3 orders of magnitude more voluminous relative to the global average.

Lower volumes and high length-to-width ratios identified near the north polar ice cap (Figure S9) might hint at a link between dune morphodynamics and volatile distribution. Areas with maximum modeled [26] annual surface CO_2 frost >40 cm (e.g., ±30°E in Figure S9a) tend to host fewer dunes, supporting the hypothesis that volatiles exert a significant control on sand mobility. By limiting sand availability, surface frost could hinder dune growth, accounting for locally less voluminous dunes [31]. Furthermore, more efficient rebound of saltating grains on a frosted bed likely increases the saltation saturation length (the spatial scale over which sand flux equilibrates to its saturated value), which in turn could result in more elongated barchan morphologies [32,33]. However, a combination of other factors could play a role in creating the observed peculiarities of barchans along the northern edge of the north polar sand sea, including complex interactions between dune fields, the atmospheric boundary layer, and polar-cap winds [34–36].

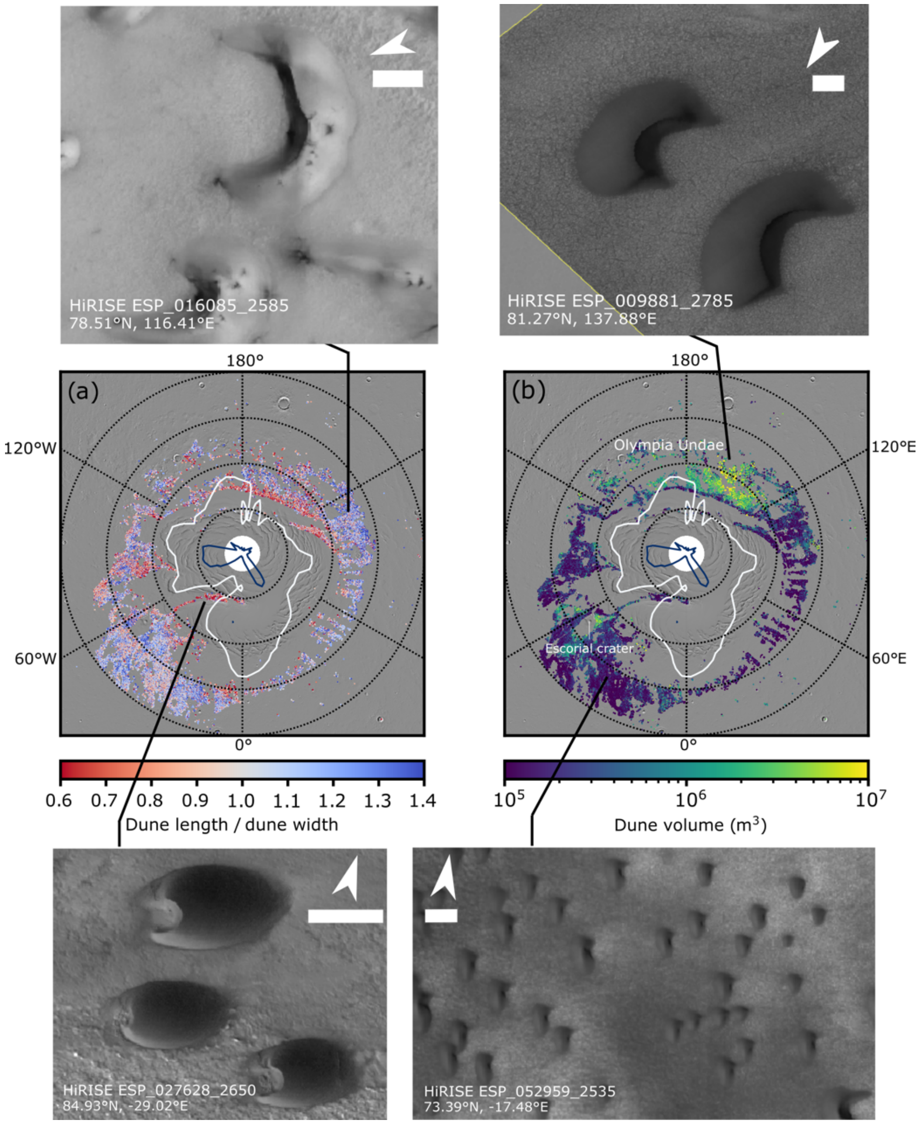

Figure S9: Maps of dune morphometrics within the martian north polar sand sea. (a) Length-to-width ratio. Contours show modeled annual maximum surface frost coverage (white = 40 cm, blue = 60 cm [26]). (b) Dune volume. HiRISE (High Resolution Imaging Experiment [5]) images show examples of various dune morphologies across the pole. Arrow indicates North, and scale bars are 100 m.

Comparison of dunes within and outside of impact craters

The effect of crater topography on the wind field and resulting eolian landforms was investigated locally, e.g., in Gale and Herschel craters [37,38], and differences in dune morphology within and outside craters have been previously noted [39]. These studies found that the concave shape of craters affects dune morphometrics in three main ways – by deflecting regional winds, by

inducing local katabatic winds [40], and by trapping sediments [41], locally increasing sediment supply. Our analysis provides quantitative evidence consistent with these effects on a global scale. We find, as predicted by models (e.g. [30]) that intracrater dunes have greater volumes and are more elongated (i.e., they have higher L/W; Figure S10). The median volume of an intracrater barchan is about 2.28×10^5 m^3, almost twice that of dunes outside of craters, ~1.45×10^5 m^3 ($p \ll 0.01$, 1-tailed t-test).

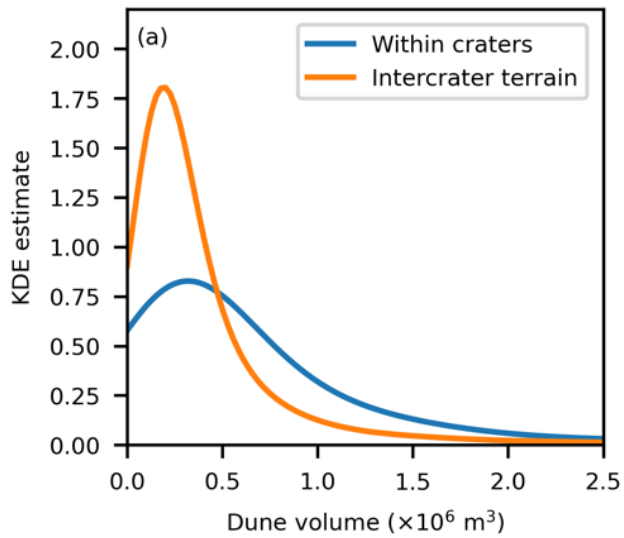

Figure S10: Barchan morphometrics inside and outside of impact craters on Mars. (A) Dune volume. (B) Dune length:width. Probability distributions are represented by their kernel density estimates (KDE). Each pair of intra- and inter-crater distributions were found to be different using a 2-tailed Kolmogorov-Smirnov test ($p<0.01$).

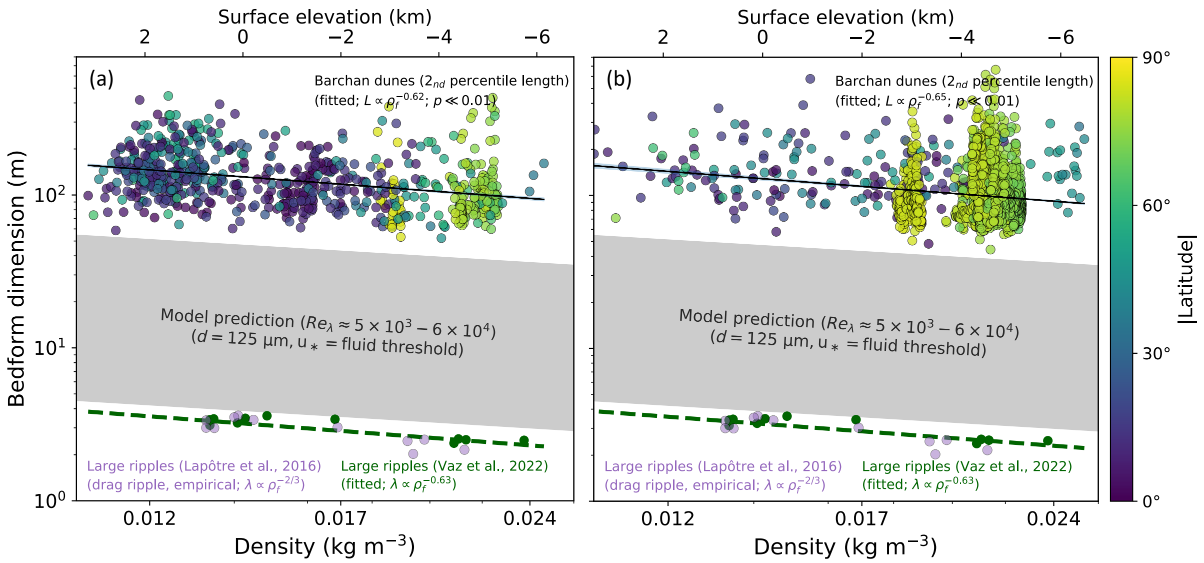

Figure S11: Same as Figure S7a, for (a) intracrater dunes and (b) intercrater dunes. Intercrater dunes show slightly higher scatter in low latitudes.

References Cited

1. Rubanenko, L., Pérez-López, S., Schull, J. & Lapôtre, M. G. A. Automatic Detection and Segmentation of Barchan Dunes on Mars and Earth Using a Convolutional Neural Network. *IEEE J. Sel. Top. Appl. Earth Obs. Remote Sens.* **14**, 9364–9371 (2021).

2. Brown, P. J. & Fuller, W. A. *Statistical analysis of measurement error models and applications: Proceedings of the AMS-IMS-SIAM joint summer research conference held June 10-16, 1989, with support from the National Science Foundation and the US Army Research Office*. vol. 112 (American Mathematical Soc., 1990).

3. Boggs, P. T., Boggs, P. T., Rogers, J. E. & Schnabel, R. B. User's reference guide for odrpack version 2.01: Software for weighted orthogonal distance regression. (1992).

4. Dickson, J. L., Kerber, L. A., Fassett, C. I. & Ehlmann, B. L. A global, blended CTX mosaic of Mars with vectorized seam mapping: A new mosaicking pipeline using principles of non-destructive image editing. in *LPSC* vol. 49 1–2 (2018).

5. McEwen, A. S. *et al.* Mars reconnaissance orbiter's high resolution imaging science experiment (HiRISE). *J. Geophys. Res. Planets* **112**, (2007).

6. Hamdan, M. A., Refaat, A. A. & Wahed, M. A. Morphologic characteristics and migration rate assessment of barchan dunes in the Southeastern Western Desert of Egypt. *Geomorphology* **257**, 57–74 (2016).

7. Yang, Z., Qian, G., Dong, Z., Tian, M. & Lu, J. Migration of barchan dunes and factors that influence migration in the Sanlongsha dune field of the northern Kumtagh Sand Sea, China. *Geomorphology* **378**, 107615 (2021).

8. Sagga, A. M. S. Barchan dunes of Wadi Khulays, western region of Saudi Arabia: geomorphology and sedimentology relationships. *Earth Sci.* **10**, (1998).

9. Sauermann, G., Rognon, P., Poliakov, A. & Herrmann, H. J. The shape of the barchan dunes of Southern Morocco. *Geomorphology* **36**, 47–62 (2000).

10. Finkel, H. J. The barchans of southern Peru. *J. Geol.* **67**, 614–647 (1959).

11. Jimenez, J. A., Maia, L. P., Serra, J. & Morais, J. Aeolian dune migration along the Ceara coast, north-eastern Brazil. *Sedimentology* **46**, 689–701 (1999).

12. Long, J. T. & Sharp, R. P. Barchan-dune movement in imperial valley, California. *Geol. Soc. Am. Bull.* **75**, 149–156 (1964).

13. Norris, R. M. Barchan dunes of Imperial Valley, California. *J. Geol.* **74**, 292–306 (1966).

14. Khalaf, F. I. & Al-Ajmi, D. Aeolian processes and sand encroachment problems in Kuwait. *Geomorphology* **6**, 111–134 (1993).

15. Sherman, D.J., Zhang, P., Bae, J., Butler, R., Rew, H. Barchan dunes: Geomorphometric data. (2021) doi:https://doi.org/10.5281/zenodo.5637339.

16. Lapôtre, M. G. A. *et al*. Large wind ripples on Mars: A record of atmospheric evolution. *Science (80-.)*. **353**, 55–58 (2016).

17. Vaz, D. A., Silvestro, S., Chojnacki, M. & Silva, D. C. A. *Global wavelength survey of Martian bedforms: methods and preliminary results*. (2022).

18. Lapôtre, M. G. A., Ewing, R. C. & Lamb, M. P. An evolving understanding of enigmatic large ripples on Mars. *J. Geophys. Res. Planets* **126**, e2020JE006729 (2021).

19. Weitz, C. M. *et al*. Sand grain sizes and shapes in eolian bedforms at Gale Crater, Mars. *Geophys. Res. Lett.* **45**, 9471–9479 (2018).

20. Andreotti, B., Claudin, P., Iversen, J. J., Merrison, J. P. & Rasmussen, K. R. A lower-than-expected saltation threshold at Martian pressure and below. *Proc. Natl. Acad. Sci.* **118**, (2021).

21. Pähtz, T., Kok, J. F. & Herrmann, H. J. The apparent roughness of a sand surface blown by wind from an analytical model of saltation. *New J. Phys.* **14**, 43035 (2012).

22. Claudin, P. & Andreotti, B. A scaling law for aeolian dunes on Mars, Venus, Earth, and for subaqueous ripples. *Earth Planet. Sci. Lett.* **252**, 30–44 (2006).

23. Kok, J. F. An improved parameterization of wind-blown sand flux on Mars that includes the effect of hysteresis. *Geophys. Res. Lett.* **37**, (2010).

24. Bagnold, R. A. *The physics of blown sand and desert dunes*. (New York, W. Morrow & company, 1942).

25. Swann, C., Sherman, D. J. & Ewing, R. C. Experimentally derived thresholds for windblown sand on Mars. *Geophys. Res. Lett.* **47**, e2019GL084484 (2020).

26. Forget, F. *et al*. Improved general circulation models of the Martian atmosphere from the surface to above 80 km. *J. Geophys. Res. Planets* **104**, 24155–24175 (1999).

27. Martin, R. L. & Kok, J. F. Wind-invariant saltation heights imply linear scaling of aeolian saltation flux with shear stress. *Sci. Adv.* **3**, e1602569 (2017).

28. Ayoub, F. *et al*. Threshold for sand mobility on Mars calibrated from seasonal variations of sand flux. *Nat. Commun.* **5**, 1–8 (2014).

29. Werner, B. T. A steady-state model of wind-blown sand transport. *J. Geol.* **98**, 1–17 (1990).

30. Parteli, E. J. R., Duran, O. & Herrmann, H. J. Minimal size of a barchan dune. *Phys. Rev. E* **75**, 11301 (2007).

31. Feldman, W. C. *et al*. Hydrogen content of sand dunes within Olympia Undae. *Icarus* **196**, 422–432 (2008).

32. Comola, F., Gaume, J., Kok, J. F. & Lehning, M. Cohesion-induced enhancement of aeolian saltation. *Geophys. Res. Lett.* **46**, 5566–5574 (2019).

33. Schatz, V., Tsoar, H., Edgett, K. S., Parteli, E. J. R. & Herrmann, H. J. Evidence for indurated sand dunes in the Martian north polar region. *J. Geophys. Res. Planets* **111**, (2006).

34. Gunn, A. *et al*. Macroscopic flow disequilibrium over aeolian dune fields. *Geophys. Res. Lett.* **47**, e2020GL088773 (2020).

35. Chojnacki, M., Banks, M. E., Fenton, L. K. & Urso, A. C. Boundary condition controls on the high-sand-flux regions of Mars. *Geology* **47**, 427–430 (2019).

36. Ewing, R. C. & Kocurek, G. A. Aeolian dune interactions and dune-field pattern formation: White Sands Dune Field, New Mexico. *Sedimentology* **57**, 1199–1219 (2010).

37. Cardinale, M. *et al*. Present-day aeolian activity in Herschel Crater, Mars. *Icarus* **265**, 139–148 (2016).

38. Baker, M. M. *et al*. The Bagnold Dunes in southern summer: Active sediment transport on Mars observed by the Curiosity rover. *Geophys. Res. Lett.* **45**, 8853–8863 (2018).

39. Bourke, M. C. & Goudie, A. S. Varieties of barchan form in the Namib Desert and on Mars. *Aeolian Res.* **1**, 45–54 (2009).

40. Fenton, L. K., Toigo, A. D. & Richardson, M. I. Aeolian processes in Proctor Crater on Mars: Mesoscale modeling of dune-forming winds. *J. Geophys. Res. Planets* **110**, (2005).

41. Gunn, A., Rubanenko, L. & Lapôtre, M. G. A. Accumulation of windblown sand in impact craters on Mars. *Geology* (2022).
